# Association Studies of Environmental Exposures, DNA Methylation and Children’s Cognitive, Behavioral, and Mental Health Problems

**DOI:** 10.3389/fgene.2022.871820

**Published:** 2022-03-31

**Authors:** Jia Guo, Kylie W. Riley, Teresa Durham, Amy E. Margolis, Shuang Wang, Frederica Perera, Julie B. Herbstman

**Affiliations:** ^1^ Columbia Center for Children’s Environmental Health, Mailman School of Public Health, Columbia University, New York, NY, United States; ^2^ Department of Biostatistics, Mailman School of Public Health, Columbia University, New York, NY, United States; ^3^ Department of Environmental Health Sciences, Mailman School of Public Health, Columbia University, New York, NY, United States; ^4^ Division of Child and Adolescent Psychiatry, Columbia University Irving Medical Center, New York, NY, United States

**Keywords:** prenatal exposure, DNA methylation, children, PM2. 5, CBCL social problems, autism, PAH

## Abstract

**Introduction:** Prenatal environmental exposures have been associated with children’s cognitive, behavioral, and mental health problems, and alterations in DNA methylation have been hypothesized as an underlying biological mechanism. However, when testing this hypothesis, it is often difficult to overcome the problem of multiple comparisons in statistical testing when evaluating a large number of developmental outcomes and DNA methylation sites as potential mediators. The objective of this study is to implement a ‘meet-in-the-middle’ approach with a sequential roadmap to address this concern.

**Methods:** In the Columbia Center for Children’s Environmental Health birth cohort study, we implemented a 5-step sequential process for identifying CpG sites that mediate associations between prenatal environmental exposures and cognitive, behavioral, and mental health problems as measured by the Wechsler Intelligence Scale for Children-Fourth Edition (WISC-IV) and the Child Behavior Checklist (CBCL). These steps include 1) the identification of biological pathways that are relevant to each outcome of interest; 2) selection of a set of genes and CpGs on genes that are significantly associated with the outcomes; 3) identification of exposures that are significantly associated with selected CpGs; 4) examination of exposure-outcome relationships among those where significant CpGs were identified; and 5) mediation analysis of the selected exposures and corresponding outcomes. In this study, we considered a spectrum of environmental exposure classes including environmental phenols, pesticides, phthalates, flame retardants and air pollutants.

**Results:** Among all considered exposures and outcomes, we found one CpG site (cg27510182) on gene (DAB1) that potentially mediates the effect of exposure to PAH on CBCL social problems at children aged 7.

**Conclusion:** This ‘meet-in-the-middle’ approach attenuates concerns regarding multiple comparisons by focusing on genes and pathways that are biologically relevant for the hypothesis.

## Introduction

Recent studies have discovered a number of associations between prenatal environmental exposures and children’s cognitive, behavioral, and mental health problems. For example, prenatal exposure to polybrominated diphenyl ether (PBDE) was found to be associated with decreased verbal performance and full-scale IQ (Herbstman et al., 2010), decreased cognitive capacity (Eskenazi et al., 2013; Chen et al., 2014), decreased language capacity (Eskenazi et al., 2013; Ding et al., 2015), decreased visual memory (Cowell et al., 2018), and decreased efficiency of the brain’s reading circuit at age 5 (Zhang et al., 2017); prenatal exposure to chlorpyrifos (CPF) has been associated with reduced full-scale IQ at ages three and 7 (Rauh et al., 2006; Rauh et al., 2011); prenatal polycyclic aromatic hydrocarbon (PAH) exposure has been linked with lower full-scale IQ, perceptual reasoning and working memory scores at age 7 (Vishnevetsky et al., 2015), and also linked with children’s lower mental development index measured by Child Behavior Checklist (CBCL) at age 3 (Perera et al., 2006), and attention-deficit/hyperactivity disorder (ADHD) behavior problems at age 9 (Perera et al., 2018), as well as alterations in the development of self-regulation capacity and social problems at age 11 (Margolis et al., 2016) and problems with inhibitory control in childhood that mediate problems with academic skills in adolescence (Margolis et al., 2021). Moreover, prenatal Bisphenol A (BPA) concentration was found to be associated with CBCL anxious/depressed and aggressive behavior (Perera et al., 2012), and CBCL internalizing and externalizing problems (Roen et al., 2015).

Environmental exposures have also been associated with epigenetic alterations including DNA methylation. Specifically, alterations in DNA methylation have been associated with exposure to BPA (Wolstenholme et al., 2011), PAH exposure and increased PAH–DNA adducts (Perera et al., 2011; Herbstman et al., 2012), exposure to phthalates (Kang and Lee, 2005), as well as exposure to high level of nitrogen dioxide (NO_2_) and fine particulate matter (PM_2.5_) (Prunicki et al., 2018). We recently developed and validated a pipeline method that predicts dichotomous high/low level of exposures such as NO_2_, PM_2.5_ and PAH using DNA methylation patterns in umbilical cord blood (Wang et al., 2021), which further demonstrated associations between DNA methylation and environmental exposures.

While previous research supports the hypotheses that prenatal exposures affect cord blood DNA methylation and also subsequent child health outcomes, it does not imply that DNA methylation is necessarily on the causal pathway between exposure and outcome. Few studies have explored the relationship among the three—environmental exposures, DNA methylation and children’s cognitive, behavioral, and mental health outcomes—simultaneously considering a spectrum of environmental exposures and a spectrum of developmental outcomes. Prior studies have focused on a single exposure or a single outcome. For example, a recent study showed that DNA methylation partly mediates the association between Bisphenol F (BPF) exposure and lower cognition in boys (Engdahl et al., 2021); another study showed that DNA methylation mediated the association between early-life lead (Pb) exposure and infant neurodevelopmental outcomes such as psychomotor development index and rating scale of emotional regulation (Rygiel et al., 2021). Similarly, another study considered a single outcome, body mass index (BMI), and a spectrum of exposures, with the goal of examining whether DNA methylation mediates the relationship between an array of environmental exposures and BMI (Cadiou et al., 2020). All of these studies were limited because they only consider a single exposure or a single outcome, because when considering many exposures or many outcomes and a large number of DNA methylation sites, the problem of multiple comparisons becomes the main limiting factor.

One study examined the effects of multiple exposures on a health outcome, BMI, via DNA methylation, by developing a “Meet-in-the-Middle” approach that attenuates the multiple comparisons problem by reducing DNA methylation dimensions *a priori*, identifying relevant genes and pathways (Cadiou et al., 2020).

In the present study, we aimed to examine the associations between a spectrum of environmental exposures and a range of children’s cognitive, behavioral, and mental health problems that might potentially be mediated through DNA methylation. We employ a similar dimension reduction approach as in (Cadiou et al., 2020) and only consider relevant biological pathways that potentially connect environmental exposures and children’s cognitive, behavioral, and mental health problems based on *a priori* knowledge obtained from the KEGG database (Kanehisa et al., 2000). We applied this approach in a longitudinal birth cohort from the Columbia Center for Children’s Environmental Health (CCCEH), seeking to identify methylation sites and corresponding genes that mediate the effect of environmental exposures on children’s cognitive, behavioral, and mental health problems.

## Materials and Methods

### Study Population

The prospective cohort study was conducted by the Columbia Center for Children’s Environmental Health, with a complete description of the study design in (Whyatt et al., 2002; Perera et al., 2002). Study subjects included 727 pregnant Dominican and African-American women recruited through local prenatal care clinics between 1998 and 2006. All women delivered at New York Presbyterian Hospital, Harlem Hospital, or their satellite clinics and were between the ages of 18–35; non-active cigarette smokers; free of diabetes, hypertension, or known HIV, having initiated prenatal care by the 20th week of pregnancy. Participants were of low-income status, with 90% of women on Medicaid.

### Exposures and Outcomes

We explored a spectrum of prenatal environmental exposures including BPA, CPF, the sum of phthalate DEHP metabolites, PBDEs, PAH, PAH-DNA adducts in maternal and cord blood, PM_2.5_, and NO_2_. For PAH and PAH adducts, we used the raw measurement, log-transformed values, and binary indicators dichotomized at the limit of detection. For PM_2.5_ and NO_2_, we used the average daily measurements within each trimester (at first, second, and third trimester separately) and average measurements across the entire pregnancy. For other exposures, we used continuous exposure measures. Detailed descriptions of exposures are reported elsewhere (Perera et al., 2016; Rauh et al., 2011; Herbstman et al., 2010; Perera et al., 2018; Factor-Litvak et al., 2014).

We considered a range of children’s cognitive, behavioral, and mental health outcomes. Cognition was measured with the Full-Scale Intelligence Quotient (FSIQ) of the Wechsler Intelligence Scale for Children-Fourth Edition (WISC-IV). The WISC-IV subtest scores were used to derive the FSIQ. Behavioral and mental health problems were measured with the Child Behavior Checklist (CBCL) and one DSM-IV oriented scale (Attention Deficit/Hyperactivity problems). Herein we analyzed WISC-IV FSIQ at age five and age 7, CBCL internalizing and externalizing composite scores at age seven and age 9, the empirically based social and attention problems syndrome scales at age seven and age 9, and the DSM-IV oriented ADHD scale at age seven and age 9. These outcomes were chosen because they have been found to be potentially associated with some environmental exposures in the literatures (Herbstman et al., 2010; Rauh et al., 2011; Perera et al., 2006; Perera et al., 2018; Margolis et al., 2016; Roen et al., 2015). Note that we considered these outcomes at different ages as separate outcomes because questionnaires that measure these outcomes at different ages might have different questions.

### DNA Methylation

DNA methylation in 432 cord blood samples was measured using the 450K array (485,577 CpG sites) and the EPIC array (866,895 CpG sites). A full description of the preprocessing and data analysis steps was included in (Wang et al., 2021). Briefly, we conducted standard quality control steps separately for 450K arrays and EPIC arrays, including requiring 95% CpG coverage per sample and 70% sample coverage per CpG, as well as removing CpGs on sex chromosomes. We also corrected for type I/II probe bias separately for two arrays using the “wateRmelon” R-package (Pidsley et al., 2013) and then combined samples with the 450K/EPIC arrays, where we kept the overlapping CpG sites that were covered by both arrays, resulting in 379,639 CpG sites. We did not perform the calibration between 450K arrays and EPIC arrays, because the BMIQ calibration method (Horvath 2013) would result in a shift between the distribution of 450K array data and the distribution of EPIC array data, while the arrays are aligned better before the calibration (Supplementary Figure S1). We used logit2 transformation to obtain M-values from methylation *β*-values, and adjusted for cell composition to obtain the M-value residuals by regressing the M-values on cell proportions, which were estimated from cord blood DNA methylation measures using the R-package “minfi” (Aryee et al., 2014). The M-value residuals were used in the following analyses.

### Statistical Analysis

Among 341 cord samples with DNA methylation data and with at least one of the outcomes considered, we randomly selected 240 samples (70%) as a discovery set and other 101 samples (30%) as a validation set. We first applied our method on the discovery set and then used the validation set to validate results.

**FIGURE 1:**
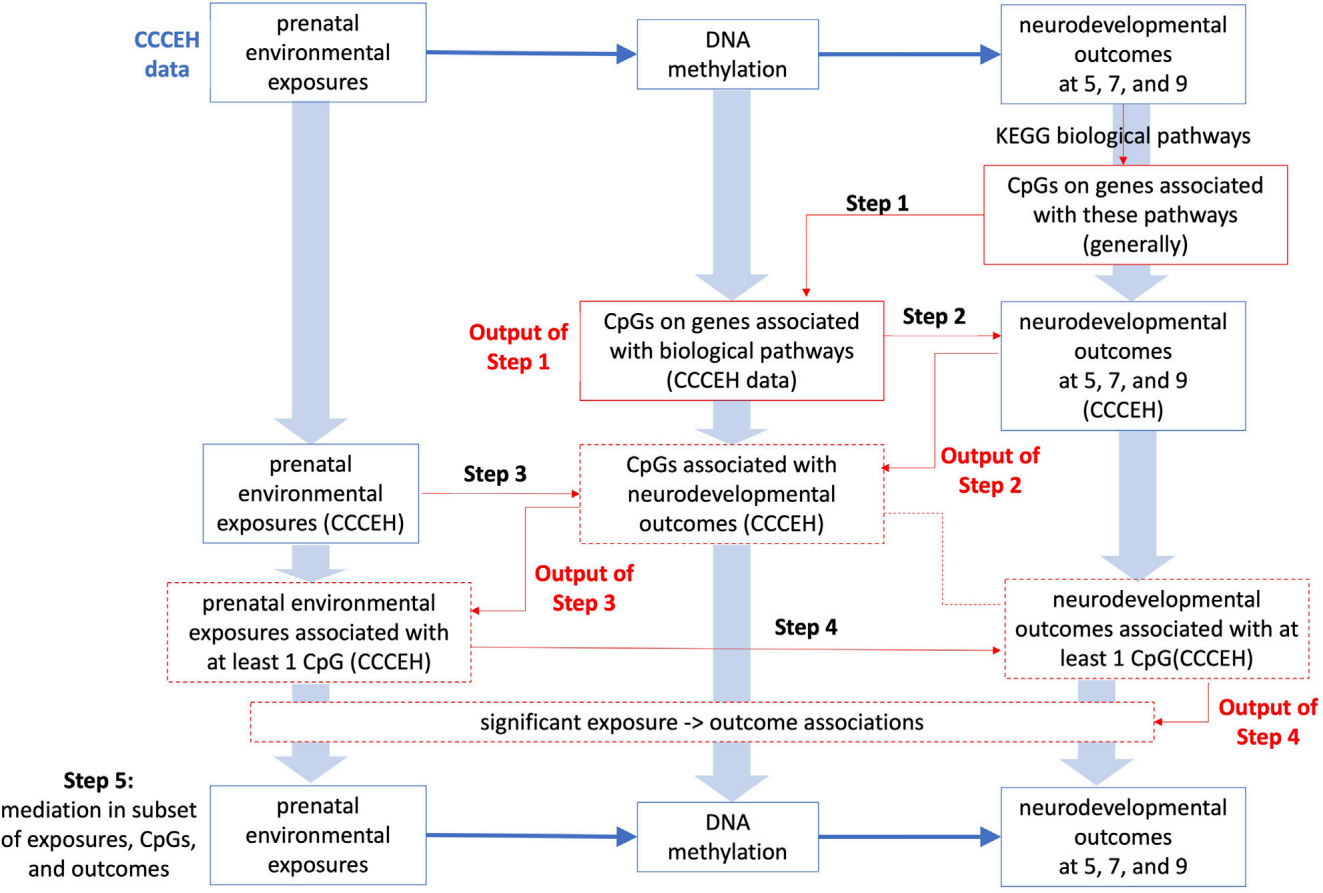
Overview of five steps.

There are five steps in our approach to investigate whether associations between prenatal environmental exposures and children’s cognitive, behavioral, and mental health problems are mediated by methylation. Before the analysis, extreme outliers (values falling outside of 4 standard deviations from the mean) are removed, where less than two samples are removed for each outcome and exposure. The steps are described in detail below and shown in Figure 1. In brief, for each outcome of interest, Step 1 selects biological pathways that are relevant to each outcome of interest based on the KEGG database, which in turn helps identify relevant genes and CpGs on these genes. Step 2 selects a set of CpGs that are significantly associated with the outcome. Step 3 identifies exposures that are significantly associated with selected CpGs from Step 2. Step 4 examines the associations between selected exposures from Step 3 and the corresponding outcome. Step 5 conducts a mediation analysis with selected CpGs and selected exposures, for each outcome of interest.

Step 1: For each neurodevelopmental outcome, we defined a set of keywords (Table 1) and then identified biological pathways related to these keywords in the KEGG database. We searched for biologically relevant pathways based on key words among all available pathways using the R-package “KEGGREST” (Tenenbaum 2016). If a keyword appears at least once in the pathway’s “Name”, “Description”, “Disease”, or titles of “Reference”, we then say the keyword is related to this pathway. A pathway is included for an outcome if this pathway is related to at least one of the predefined keywords. Examples of identified pathways related to the keyword “depression” can be found in the Supplementary material SA. We tried a large set of keywords (Supplementary material SB), and in Table 1 we only showed the keywords that are related to at least one biological pathway. For each outcome, we then extracted genes in the identified pathways and CpGs on these genes.

**TABLE 1:** Keywords for each of children’s neurodevelopmental outcome.

Cognitive, Behavioral, and Mental Health Outcomes Considered	Keywords
IQ full score at age 5	children, brain, neuron, intelligent, intelligence, IQ
IQ full score at age 7	children, brain, neuron, intelligent, intelligence, IQ
CBCL internal at age 7	children, brain, neuron, depressed, depression, depressive
CBCL internal at age 9	children, brain, neuron, depressed, depression, depressive
CBCL external at age 7	children, brain, neuron, aggression, aggressive
CBCL external at age 9	children, brain, neuron, aggression, aggressive
CBCL social problem at age 7	children, brain, neuron, autism, social
CBCL social problem at age 9	children, brain, neuron, autism, social
CBCL attention problem at age 7	children, brain, neuron, attention, hyperactivity
CBCL attention problem at age 9	children, brain, neuron, attention, hyperactivity
DSM ADHD at age 7	children, brain, neuron, attention, hyperactivity
DSM ADHD at age 9	children, brain, neuron, attention, hyperactivity

Step 2: For each outcome, we tested its associations with each of the preselected CpGs in Step 1 using regression models, adjusting for children’s sex (male vs. female), ethnicity (Dominican vs. African American), and children’s age of testing when outcomes are measured (Eqs 1, 2, where ${\text{Y}}_{\text{i}}$ denoted the *i*th outcome and ${\text{CpG}}_{\text{k}}$ denoted the M-value residual of the *k*th CpG). We used linear regression models for the IQ full scores, and negative binomial regression models for CBCL subscales and DSM ADHD outcomes. We included the ages of testing because it might not be exactly seven or 9, with up to a few months shifted. For each outcome, we applied false discovery rate (FDR) with adjusted-p<0.05 as the threshold to correct for multiple comparisons.

$$\begin{array}{c} \text{E}\left({\text{Y}}_{\text{i}}{\text{|CpG}}_{\text{k}},\;\text{c}\text{o}\text{v}\text{s}\right)={\text{β}}_{0}+{\text{β}}_{1}{\text{CpG}}_{\text{k}}+{\text{β}}_{2}I\left(\text{Sex}=\text{male}\right)+{\text{β}}_{3}I\left(\text{Ethnicity}=\text{Dominican}\right)+{\text{β}}_{4}\text{A}\text{g}\text{e} \end{array}$$ (1)

$$\begin{array}{c} \text{l}\text{o}\text{g}\left(\text{E}\left({\text{Y}}_{\text{i}}{\text{|CpG}}_{\text{k}},\text{covs}\right)\right)={\text{β}}_{0}+{\text{β}}_{1}{\text{CpG}}_{\text{k}}+{\text{β}}_{2}I\left(\text{Sex}=\text{male}\right)+{\text{β}}_{3}I\left(\text{Ethnicity}=\text{Dominican}\right)+{\text{β}}_{4}Age \end{array}$$ (2)

**Step 3**: For each outcome, we tested associations between each of the environmental exposures and each of the significant CpGs identified in Step 2, adjusting for children’s sex and ethnicity (Eq. 3, where ${\text{X}}_{\text{j}}$ is the *j*th exposure, ${\text{CpG}}_{\text{k}}$ is the M-value residual of the *k*th CpG). For each outcome, we used FDR to adjust for multiple comparisons (number of test = number of exposure * number of significant CpG identified in Step 2). For each outcome, this step selected a set of exposures that were associated with CpGs among the significant CpGs selected in Step 2. Note that this step requires samples to have outcome measures, methylation data and exposures data.

$$\begin{array}{c} \text{E}\left({\text{CpG}}_{\text{k}}{\text{|X}}_{\text{j}},\;\text{c}\text{o}\text{v}\text{s}\right)={\text{β}}_{0}+{\text{β}}_{1}{\text{X}}_{\text{j}}+{\text{β}}_{2}I\left(\text{Sex}=\text{male}\right)+{\text{β}}_{3}I\left(\text{Ethnicity}=\text{Dominican}\right) \end{array}$$ (3)

Step 4: For each outcome, we tested associations with environmental exposures selected in Step 3, adjusting for children’s sex, ethnicity, and children’s age of testing (Eqs 4, 5). We used FDR to adjust for multiple comparisons (number of tests = number of outcomes ∗ number of exposures).

$$\begin{array}{c} \text{E}\left({\text{Y}}_{\text{i}}{\text{|X}}_{\text{j}},\;\text{c}\text{o}\text{v}\text{s}\right)={\text{β}}_{0}+{\text{β}}_{1}{\text{X}}_{\text{j}}+{\text{β}}_{2}I\left(\text{Sex}=\text{male}\right)+{\text{β}}_{3}I\left(\text{Ethnicity}=\text{Dominican}\right)+{\text{β}}_{4}\text{A}\text{g}\text{e} \end{array}$$ (4)

$$\begin{array}{c} \text{l}\text{o}\text{g}\left(\text{E}\left({\text{Y}}_{\text{i}}{\text{|X}}_{\text{j}},\text{covs}\right)\right)={\text{β}}_{0}+{\text{β}}_{1}{\text{X}}_{\text{j}}+{\text{β}}_{2}I\left(\text{Sex}=\text{male}\right)+{\text{β}}_{3}I\left(\text{Ethnicity}=\text{Dominican}\right)+{\text{β}}_{4}\text{A}\text{g}\text{e} \end{array}$$ (5)

Step 5: For each exposure-outcome pair with a significant association identified in Step 4, we conducted mediation analysis using the R-package ‘MMA’, where mediators are CpGs that are associated with both the outcome (Step 2) and the exposure (Step 3). The ‘MMA’ package uses a bootstrap sampling method to estimate indirect and direct effects of the exposure on the outcome. The indirect effect measures the extent to which the exposure influences the outcome through CpGs, while the direct effect constitutes the extent to which the exposure directly influences the outcome without CpGs. We reported the percentage of the mediation effect in the total effect, calculated as in Eq. 6:

$$\begin{array}{c} p=\frac{abs\left(\text{indirect effect }\right)}{abs\left(\text{indirect effect }\right)+abs\left(\text{direct effect }\right)} \end{array}$$ (6)

## Results

Table 2 shows the numbers of pathways, genes and CpGs that are selected from KEGG database using keywords for different outcomes (from Step 1). Table 2 also shows the number of CpGs (from Step 2) that are significantly associated with each outcome in the CCCEH dataset after FDR adjustment for multiple testing. CBCL internalizing problems at age nine and CBCL social problems at age seven were found to be associated with two preselected CpGs after FDR adjustment for multiple comparisons, while no CpGs were found to be significantly associated with other outcomes. Full results of Step 2 were showed in Supplementary Figure S2; Supplementary Material SC.

**TABLE 2:** Numbers of selected pathways/genes/CpGs based on keywords and numbers of CpGs that are significantly associated with outcomes (Output of Step 1 and Step 2).

Cognitive, Behavioral, and Mental Health Outcomes Considered	Output of step 1				Output of step 2
	# Pathways	# Genes	# Illumina Annotated Genes out of Pathway Identified Genes	# CpGs	# Significant CpGs*
IQ full scale at age 5	42	2,805	2,376	40,864	0
IQ full scale at age 7	42	2,805	2,376	40,864	0
CBCL internalizing problems at age 7	44	2,792	2,364	41,002	0
CBCL internalizing problems at age 9	44	2,792	2,364	41,002	2
CBCL externalizing problems at age 7	47	2,860	2,429	42,182	0
CBCL externalizing problems at age 9	47	2,860	2,429	42,182	0
CBCL social problems at age 7	44	2,846	2,415	42,229	2
CBCL social problems at age 9	44	2,846	2,415	42,229	0
CBCL attention problems at age 7	43	2,947	2,507	42,512	0
CBCL attention problems at age 9	43	2,947	2,507	42,512	0
DSM ADHD at age 7	43	2,947	2,507	42,512	0
DSM ADHD at age 9	43	2,947	2,507	42,512	0

*at *p* < 0.05 after accounting for multiple comparisons using FDR, correction.

For each outcome identified in Step 2 (Table 2) with at least one associated CpG site, Step 3 examines the relationship between each environmental exposure and the subset of CpGs identified in Step 2. Table 3 lists the exposures that are significantly associated with at least one CpG after FDR adjustment for multiple comparisons, representing the output of Step 3. For the two CpGs (cg27510182 and cg24713878) that were associated with CBCL social problems at age 7, only CpG cg27510182 is significantly associated with log-transformed PAH after accounting for multiple comparisons using FDR. No exposures were found to be significantly associated with these two CpGs that are associated with CBCL internalizing problems at age 9. Full results of Step 3 were included in Supplementary Material SD.

**TABLE 3:** Selected exposures for each outcome and the number of CpGs that are significantly associated with the exposures (Output of Step 3).

Selected Cognitive, Behavioral, and Mental Health Outcomes	# CpGs Considered	Exposures	# Significant CpGs*	CpG	Raw P	FDR Adj. P
CBCL social problems at age 7	2	log-transformed PAH	1	cg27510182	0.0008	0.0325
				cg24713878	0.0313	0.2194
		Other Exposures	0	\	\	\
CBCL internalizing problems at age 9	2	Other Exposures	0	\	\	\

*at *p* < 0.05 after accounting for multiple comparisons using FDR, correction.

For the exposure and outcomes that show significant findings in Step 3, Step 4 evaluates the exposure-outcome relationships. The only association needs to be tested is the association between CBCL social problems at age seven and log-transformed PAH. Table 4 shows that the log-transformed PAH is positively and significantly associated with CBCL social problems at age 7. The direction is expected because higher scores of CBCL indicates more problems.

**TABLE 4:** Association tests between outcome and selected exposures which were significantly associated with some outcome-related CpGs (Output from Step 4).

Selected Cognitive, Behavioral, and Mental Health Outcomes	Selected Exposures	Estimate	Raw P
CBCL social problems at age 7	log-transformed PAH	0.2386	0.0291

For the significant exposure-outcome relationship of Step 4, Step 5 evaluates the percentage of mediation effects in the total effect, when those potential CpGs are found to be mediators (Table 5). For the effect of log-transformed PAH on CBCL social problems at age 7, cg27510182 has a mediation effect about 46.7% of the total effect. In Table 6, we summarized our overall finding in the discovery dataset with 240 samples with cg27510182, including regression coefficients and raw *p*-values for each step that involve this CpG from CpG-outcome, exposure-CpG and exposure-outcome relationships. The corresponding results in the validation dataset with 101 samples are included in Supplementary Table S1, where the associations were not significant, possibly due to the small sample size, but the directions of CpG-outcome and exposure-CpG relationships were replicated as those in the discovery dataset.

**TABLE 5:** Mediation analysis of selected paths of exposure-CpG-outcome (Results from Step 5).

Selected Exposures	CpG	Selected Cognitive, Behavioral, and Mental Health Outcomes	Percentage of Mediation Effect in Total Effect (%)
log-transformed PAH	cg27510182	CBCL social problems at age 7	46.7

**TABLE 6:** Overall findings of CpG cg27510182 from each step in the discovery dataset with 240 samples.

Steps	Dependent Variables	Independent Variables	Estimate	Raw P
Step 2*	CBCL social problems at age 7	cg27510182	0.5807	<0.0001
Step 3**	cg27510182	log-transformed PAH	0.2333	0.0008
Step 4*	CBCL social problems at age 7	log-transformed PAH	0.2386	0.0291

*Negative binomial regression adjusting for sex, ethnicity, and age at testing

**Linear regression adjusting for sex and ethnicity.

We further summarized the identified CpG cg27510182 and the corresponding gene together with their related keywords and pathways from the KEGG database in Table 7.

**TABLE 7:** Identified genes with related keywords and pathways from KEGG database.

CpG	CHR	Mapinfo	Gene	KEGG Pathways	Related Keywords
cg27510182	1	58715553	DAB1 (DAB Adaptor Protein 1)	Spinocerebellar ataxia (hsa05017)	Neuron

## Discussion

In the CCCEH birth cohort, we have explored the associations between a spectrum of environmental exposures, DNA methylation, and a range of children’s cognitive, behavioral, and mental health problems using a “Meet-in-the-Middle” approach. As previously noted, this approach takes advantage of relevant biological pathways to initially reduce the number of DNA methylation CpG sites tested. Using sequential steps that further reduce the number of comparisons, this methodology can be used to test exposure-DNA methylation-outcome relationships where *a priori* information supports the biological plausibility of findings. To compare with our method, we also conducted a standard EWAS (epigenome-wide association study) for each considered outcome, without using relevant biological pathways to preselect CpGs. Although we found CpGs that are significantly associated with some outcomes (Supplementary Material SE) in EWAS after FDR adjustment, there was no significant finding from the following steps based on these significant CpGs. This emphasizes the benefit of our method.

Among all the exposure-DNA methylation-outcome relationships we examined, we found one CpG site and one gene that potentially mediates the effect of exposure to PAH on CBCL social problems at age 7. Specifically, we identified the methylation CpG cg27510182 and corresponding gene DAB1 (DAB Adaptor Protein 1), that potentially mediate the effect of exposure to PAH on CBCL social problems. Close investigation of the identified gene and its relevant pathways suggests that they are biologically relevant. The identified gene DAB1 has been reported to be associated with many neurodevelopmental and psychiatric disorders, such as schizophrenia (SCZ) and autism spectrum disorders (ASD) (Wang et al., 2014; Li et al., 2015; Chen et al., 2017; Stessman et al., 2017; Sánchez-Sánchez et al., 2018; Nawa et al., 2020), because DAB1 is involved in the Reelin signaling pathway which plays a critical role in the central nervous system such as regulating neuronal position in the developing brain (Howell et al., 1997; Rice et al., 2001; Trotter et al., 2013). In addition, the identified gene DAB1 is in the KEGG pathway of a group of progressive neurodegenerative diseases “Spinocerebellar ataxia” (hsa05017), which are usually due to the dysfunction of the cerebellum (Paulson 2009; Matilla-Dueñas et al., 2010), and it has been reported that cerebellar damage is associated with an increased risk of the ASD (Becker et al., 2013; D'Mello et al., 2015). Thus, prenatal exposure to PAH may associate with the malfunction of cerebellum and the Reelin signaling pathway via epigenetic processes, which then associate with children’s neurodevelopmental problems, such as the ASD. In conclusion, the Meet-in-the-Middle approach has revealed that DNA methylation may mediate the effect of prenatal exposure of PAH on neurodevelopmental problems, by affecting the potentially relevant portion of the brain and neurological pathways.

There have been many studies focusing on the relationships between environmental exposures and neurodevelopmental outcomes through DNA methylation. Some studies considered one single exposure and one single outcome (Engdahl et al., 2021); some studies considered one single exposure and multiple outcomes (Rygiel et al., 2021); while some studies considered multiple exposures and one single outcome (Cadiou et al., 2020). Our research is distinct from these existing studies in that we simultaneously consider a spectrum of exposures and a spectrum of correlated cognitive, behavioral, and mental health problems outcomes. To overcome the multiple comparison problem in our work with many exposures and many outcomes as well as high dimensional DNA methylation, we identified a subset of genes in relevant biological pathways with neurodevelopmental outcomes through keywords search.

The step of keywords search is also a limitation of our study, as different keywords will identify different subsets of genes, which may lead to different methylation mediation effects. A more rigorous and more specific method to choose the keywords is necessary. Another limitation is that the identified biological pathways and genes are obtained from a single database, KEGG, which may only contain limited knowledge from existing literature. Besides, the relatively small sample size and lack of adjusting other potential confounders are also limitations of our study. In this study, we randomly selected 70% of our samples as the discovery set and the other 30% as the validation set, which further reduces the sample sizes in each set. We acknowledge the randomness in sample selection, which may affect the findings in both discovery set and the validation set. However, we want to emphasize that we our method is promising and can overcome small sample size problems when large prospective birth cohort studies with multiple cognitive, behavioral, and mental health outcomes of children and multiple prenatal environmental exposures, together with epigenetics are relatively hard to find.

In summary, the methodology outlined in this analysis provides a roadmap for analyses that reserve hypothesis testing to relationships along the exposure-DNA methylation-outcome pathway that have enhanced biological plausibility and thus increased the potential to provide meaningful results. In this study, we detected one CpG cg27510182 on the gene *DAB1* that partially mediates the association between prenatal exposure to PAH and CBCL social problems at ages 7. The results are consistent with most recent literature, where PAH could produce a long-lasting effect on self-regulatory capacities and PAH-DNA adduct had a positive association with the CBCL social competence problem (Margolis et al., 2016). PAH exposure during pregnancy has been reported to be positively correlated with CBCL social scores and also positively correlated with the Autism Behavior Checklist (ABC) total scores, which suggest that PAH could be one of the risk factors of ASD-related behaviors for children (Liu et al., 2019). Future epidemiologic studies that can replicate/confirm these associations and mechanistic studies that can evaluate these pathways will enhance our understanding of how prenatal exposure of PAH may lead to neurodevelopmental problems in children.

## Data Availability

The raw data supporting the conclusion of this article will be made available by the authors, without undue reservation.
