# Supplementary material for: Association Studies of Environmental Exposures, DNA Methylation and Children’s Cognitive, Behavioral, and Mental Health Problems
Source: Front Genet. 2022 Mar 31;13:871820. doi: 10.3389/fgene.2022.871820 (PMC9074894; doi:10.3389/fgene.2022.871820)
Supplement: Supplementary file 1 [file DataSheet1.zip › Supplementary Materials/Supplement_Figure_S1.pdf]

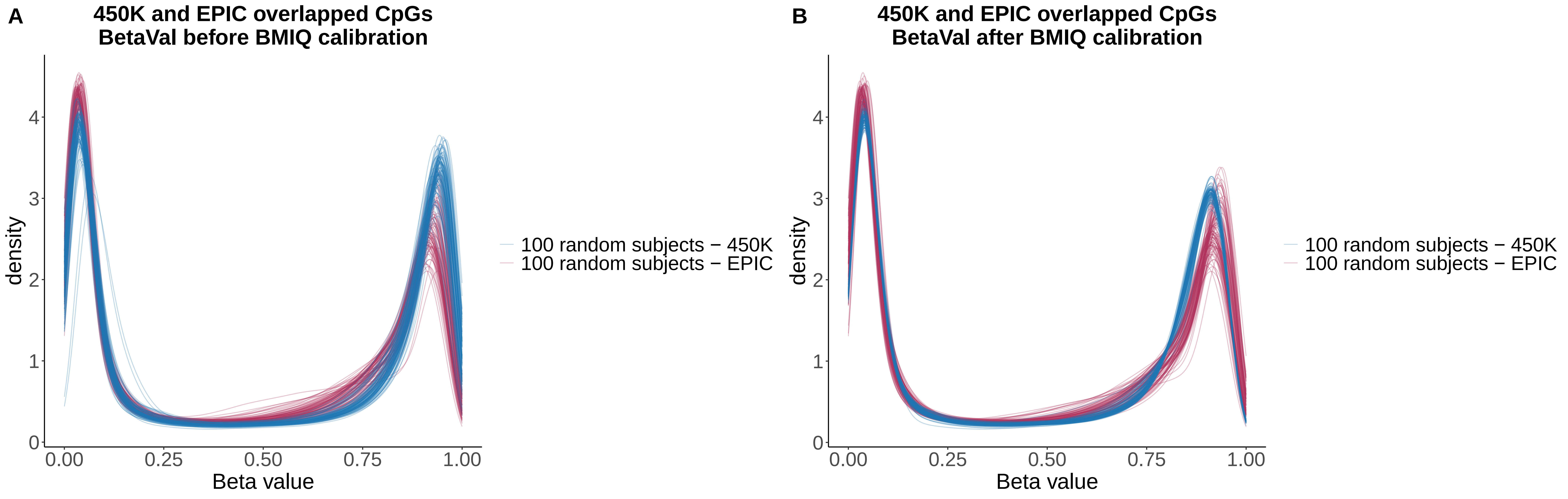
A
450K and EPIC overlapped CpGs
BetaVal before BMIQ calibration
density
Beta value
0.00
0.25
0.50
0.75
1.00
0
1
2
3
4
100 random subjects − 450K
100 random subjects − EPIC
B
450K and EPIC overlapped CpGs
BetaVal after BMIQ calibration
density
Beta value
0.00
0.25
0.50
0.75
1.00
0
1
2
3
4
100 random subjects − 450K
100 random subjects − EPIC
