# Supplementary material for: Association Studies of Environmental Exposures, DNA Methylation and Children’s Cognitive, Behavioral, and Mental Health Problems
Source: Front Genet. 2022 Mar 31;13:871820. doi: 10.3389/fgene.2022.871820 (PMC9074894; doi:10.3389/fgene.2022.871820)
Supplement: Supplementary file 1 [file DataSheet1.zip › Supplementary Materials/Supplement_Table_S1.docx]

**Table S1. Overall findings of CpG cg27510182 from each step in the validation dataset with 101 samples.**

| Steps | Dependent variables | Independent variables | Estimate | Raw P |
| --- | --- | --- | --- | --- |
| Step 2* | CBCL social problems at age 7 | cg27510182 | 0.1361 | 0.3496 |
| Step 3** | cg27510182 | log-transformed PAH | 0.1026 | 0.3965 |
| Step 4* | CBCL social problems at age 7 | log-transformed PAH | -0.0221 | 0.8871 |

* Negative binomial regression adjusting for sex, ethnicity, and age at testing

** Linear regression adjusting for sex and ethnicity
