# Supplementary material for: Association Studies of Environmental Exposures, DNA Methylation and Children’s Cognitive, Behavioral, and Mental Health Problems
Source: Front Genet. 2022 Mar 31;13:871820. doi: 10.3389/fgene.2022.871820 (PMC9074894; doi:10.3389/fgene.2022.871820)
Supplement: Supplementary file 1 [file DataSheet1.zip › Supplementary Materials/Supplement_SA_pathway_example.docx]

**Association studies of environmental exposures, DNA methylation and children’s cognitive, behavioral, and mental health problems**

**Supplementary Materials A**

Jia Guo^ab^, Kylie W. Riley^ac^, Teresa Durham^ac^, Amy Margolis^ac^, Shuang Wang^ab^, Frederica Perera^ac^, Julie B. Herbstman^ac^

^a^ Columbia Center for Children’s Environmental Health, Mailman School of Public Health, Columbia University, New York, New York

^b^ Department of Biostatistics, Mailman School of Public Health, Columbia University, New York, New York

^c^ Department of Environmental Health Sciences, Mailman School of Public Health, Columbia University, New York, New York

**Two identified pathways related to the keyword “depression”**

The first pathway is hsa04730, *Long-term depression - Homo sapiens (human)*, which is matching the keyword “depression” because “depression” appears in its name, description, and the titles of its references.

The second pathway is hsa04723, *Retrograde endocannabinoid signaling - Homo sapiens (human)*, which is matching the keyword “depression” because “depression” appears in the title of one of its references.

Details of each pathway obtained from the R-package “KEGGREST” are showed below. Only part of results are showed due to limited space.

**Pathway hsa04730**

Name: Long-term **depression** - Homo sapiens (human)

Description: Cerebellar long-term **depression** (LTD), thought to be a molecular and cellular basis for cerebellar learning, is a process involving a decrease in the synaptic strength between parallel fiber (PF) and Purkinje cells (PCs) induced by the conjunctive activation of PFs and climbing fiber (CF). Multiple signal transduction pathways have been shown to be involved in this process. Activation of PFs terminating on spines in dendritic branchlets leads to glutamate release and activation of both AMPA and mGluRs. Activation of CFs, which make multiple synaptic contacts on proximal dendrites, also via AMPA receptors, opens voltage-gated calcium channels (VGCCs) and causes a generalized influx of calcium. These cellular signals, generated from two different synaptic origins, trigger a cascade of events culminating in a phosphorylation-dependent, long-term reduction in AMPA receptor sensitivity at the PF-PC synapse. This may take place either through receptor internalization and/or through receptor desensitization.

Class: Organismal Systems; Nervous system

Disease: Spinocerebellar ataxia (SCA), Central core disease, Episodic ataxias, etc.

Organism: Homo sapiens (human)

Gene: NOS1, GUCY1A2, GUCY1A1, GUCY1B1, PRKG1, etc.

Compound: L-Glutamate, Calcium cation, Diacylglycerol, Arachidonate, etc.

Reference:

[1] Ito, M. (2002). The molecular organization of cerebellar long-term **depression**. Nature Reviews Neuroscience, 3(11), 896-902.

[2] Ito, M. (2001). Cerebellar long-term **depression**: characterization, signal transduction, and functional roles. Physiological reviews, 81(3), 1143-1195.

[3] Purves, D. G., Fitzpatrick, A. D., Katz, L. C., La Mantia, A. S., & McNamara, J. O. (1997). Neuroscience. Sunderland Mass: Sinauer Assoc. Inc. Publ, 121-44.

[4] Daniel, H., Levenes, C., & Crépel, F. (1998). Cellular mechanisms of cerebellar LTD. Trends in neurosciences, 21(9), 401-407.

[5] Levenes, C., Daniel, H., & Crépel, F. (1998). Long-term **depression** of synaptic transmission in the cerebellum: cellular and molecular mechanisms revisited. Progress in neurobiology, 55(1), 79-91.

[6] Metzger, F., & Kapfhammer, J. P. (2003). Protein kinase C: its role in activity-dependent Purkinje cell dendritic development and plasticity. The cerebellum, 2(3), 206-214.

**Pathway hsa04723**

Name: Retrograde endocannabinoid signaling - Homo sapiens (human)

Description: Endogenous cannabinoids (endocannabinoids) serve as retrograde messengers at synapses in various regions of the brain. The family of endocannabinoids includes at least five derivatives of arachidonic acid; the two best characterized are arachydonoyl ethanolamide (anandamide, AEA) and 2-arachydonoil glycerol (2AG). They are released from postsynaptic neurons upon postsynaptic depolarization and/or receptor activation. The released endocannabinoids then activate the CB1 receptors (CB1R) at presynaptic terminals and suppress the release of inhibitory transmitter GABA (depolarization-induced suppression of inhibition, DSI) or excitatory transmitter glutamate (depolarization-induced suppression of excitation, DSE) by inhibiting Ca2+ channels. Besides the well-known expression of the CB1R in the plasma membrane, this receptor is also present in mitochondrial membranes, where it reduces the mitochondrial respiration and contributes to DSI. Whereas DSI and DSE result in short-term synaptic plasticity, endocannabinoids also mediate long-term synaptic changes (eCB-LTD). Persistent activation of CB1 receptors over a period of minutes triggers eCB-LTD by a RIM1alpha-dependent mechanism.

Class: Organismal Systems; Nervous system

Disease: Early infantile epileptic encephalopathy

Drug: Levonantradol hydrochloride (USAN), Nantradol hydrochloride (USAN), etc.

Organism: Homo sapiens (human)

Gene: SLC17A6, SLC17A8, SLC17A7, GRIA1, GRIA2, GRIA3, etc.

Compound: L-Glutamate, Calcium cation, Glycerol, Phosphatidylcholine, etc.

Reference:

[1] Hashimotodani, Y., Ohno-Shosaku, T., & Kano, M. (2007). Ca2+-assisted receptor-driven endocannabinoid release: mechanisms that associate presynaptic and postsynaptic activities. Current opinion in neurobiology, 17(3), 360-365.

[2] Ohno-Shosaku, T., Tanimura, A., Hashimotodani, Y., & Kano, M. (2012). Endocannabinoids and retrograde modulation of synaptic transmission. The Neuroscientist, 18(2), 119-132.

…

[16] Lafourcade, C. A. (2009). Presynaptic mechanisms of endocannabinoid-mediated long-term **depression** in the hippocampus. Journal of neurophysiology, 102(4), 2009-2012.

…

[21] Harkany, T., & Horvath, T. L. (2017). (S) Pot on mitochondria: cannabinoids disrupt cellular respiration to limit neuronal activity. Cell Metabolism, 25(1), 8-10.

[22] Alger, B. E., & Tang, A. H. (2012). Do cannabinoids reduce brain power?. Nature neuroscience, 15(4), 499-501.
