# Supplementary figures and images for: Association Studies of Environmental Exposures, DNA Methylation and Children’s Cognitive, Behavioral, and Mental Health Problems
Source: Front Genet. 2022 Mar 31;13:871820. doi: 10.3389/fgene.2022.871820 (PMC9074894; doi:10.3389/fgene.2022.871820)

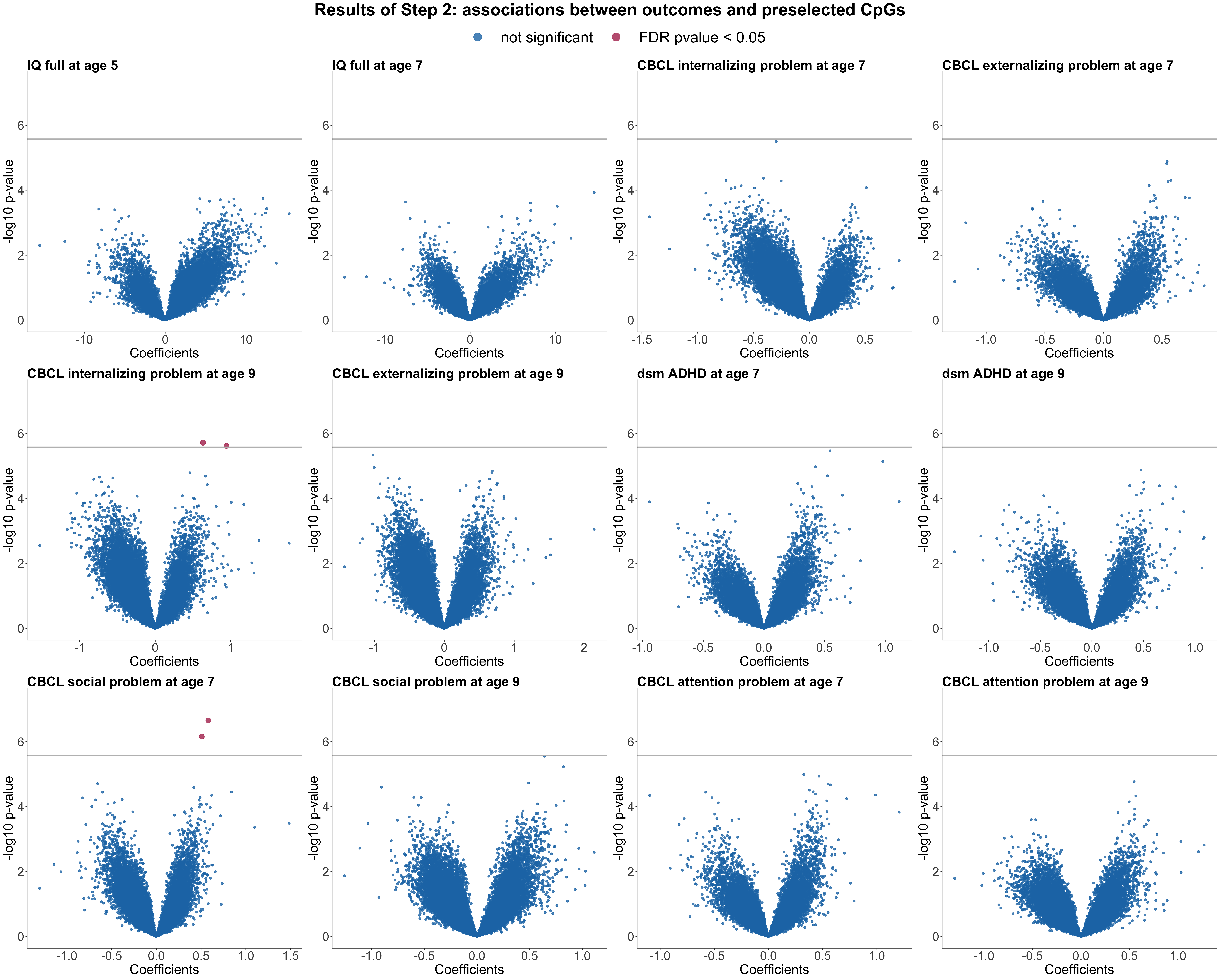

Supplement: Supplementary file 1 [file DataSheet1.zip › Supplementary Materials/Supplement_Figure_S2.png]
